# Modulation of spatial and temporal modules in lower limb muscle activations during walking with simulated reduced gravity

**DOI:** 10.1038/s41598-021-94201-9

**Published:** 2021-07-20

**Authors:** Shota Hagio, Makoto Nakazato, Motoki Kouzaki

**Affiliations:** 1 Laboratory of Neurophysiology, Graduate School of Human and Environmental Studies, Kyoto University, Yoshida-nihonmatsu-cho, Sakyo-ku, Kyoto, 606-8501 Japan; 2 Unit of Synergetic Studies for Space, Kyoto University, Kyoto, 606-8502 Japan

**Keywords:** Neuroscience, Motor control

## Abstract

Gravity plays a crucial role in shaping patterned locomotor output to maintain dynamic stability during locomotion. The present study aimed to clarify the gravity-dependent regulation of modules that organize multiple muscle activities during walking in humans. Participants walked on a treadmill at seven speeds (1–6 km h^−1^ and a subject- and gravity-specific speed determined by the Froude number (*Fr*) corresponding to 0.25) while their body weight was partially supported by a lift to simulate walking with five levels of gravity conditions from 0.07 to 1 g. Modules, i.e., muscle-weighting vectors (spatial modules) and phase-dependent activation coefficients (temporal modules), were extracted from 12 lower-limb electromyographic (EMG) activities in each gravity (*Fr* ~ 0.25) using nonnegative matrix factorization. Additionally, a tensor decomposition model was fit to the EMG data to quantify variables depending on the gravity conditions and walking speed with prescribed spatial and temporal modules. The results demonstrated that muscle activity could be explained by four modules from 1 to 0.16 g and three modules at 0.07 g, and the modules were shared for both spatial and temporal components among the gravity conditions. The task-dependent variables of the modules acting on the supporting phase linearly decreased with decreasing gravity, whereas that of the module contributing to activation prior to foot contact showed nonlinear U-shaped modulation. Moreover, the profiles of the gravity-dependent modulation changed as a function of walking speed. In conclusion, reduced gravity walking was achieved by regulating the contribution of prescribed spatial and temporal coordination in muscle activities.

## Introduction

All whole-body movements on Earth, such as upright posture and locomotion in humans, are strongly determined by the gravitational force equal to 1 g^1,2^. Since being born in these terrestrial circumstances, the nervous system has been organized to encode the gravity-related sensory information^3^. In addition, progress in space science has enabled the human race to challenge space exploration to the moon or Mars, which involves a sudden transition between different gravity conditions. These challenges require rapid movement corrections, such as for preventing a fall during locomotion, in novel gravitational environments by recalibrating gravity-related sensorimotor transformation^4,5^. However, questions still exist regarding the modulation of neuromuscular control depending on gravity.

Gravitational load plays a crucial role in shaping patterned motor output during walking in humans^6–9^. Walking under reduced gravity has been frequently simulated with a body weight support (BWS) system and parabolic flight, revealing that the walking is mechanically impeded as gravity decreases^10,11^. The external mechanical work is reduced by low gravity, mostly due to the reduction in potential energy of the body centre of mass^12–15^. This reduction negatively affects the pendulum-like saving mechanism of walking based on the exchange of potential and kinetic energy to minimize muscular work. Moreover, low gravity also changes the molecular, cellular and neuromuscular mechanisms of the nervous system that underlie the control of walking movement^16^. These effects of low gravity on the mechanical and physiological aspects change muscle activity, which is an important parameter for determining a preferred gait pattern in a low gravity environment^17^. For instance, the amplitude of activity in the ankle extensors systematically decreases with decreasing simulated gravity, consistent with their antigravity function^6,18,19^. By contrast, the quadriceps muscles demonstrated an increasing amplitude of their activity, which could not be predicted simply based on the static load during stance^19^. Thus, the locomotor system characterizes individual muscle activations during walking under low-gravity conditions, while how the muscles are coordinated depending on gravity levels remains not fully understood.

The use of a computational decomposition technique has revealed that the movement-related complex activation patterns in multiple muscles could be captured by the combination of a small number of modules, i.e., groups of muscles^20–24^. Electromyographic (EMG) activity during locomotion can be decomposed into several sets of weighting coefficients on individual muscles, i.e., spatial modules, and activation coefficients related to the phase of locomotion, i.e., temporal modules, in each module^25–27^. Previous studies have reported that a wide range of muscle activation in locomotor-like movements, such as walking, running and obstacle clearance, can be explained by shared spatial and temporal modules with several movement-dependent modules^25,28–30^. A study in walking with body weight unloading demonstrated that the temporal modules shared by multiple muscles were similar across each simulated gravity condition between 1 and 0.05 g, whereas the spatial modules were considerably different among conditions^31^. Furthermore, individual muscle activities, especially in thigh muscles, also showed large variability across participants with BWS, which may lead to large inter-subject variability of extracted modules^19,32^. Accordingly, how the spatial and temporal locomotor modules are modulated as a function of gravity has not been systematically elucidated.

**Figure 1:**
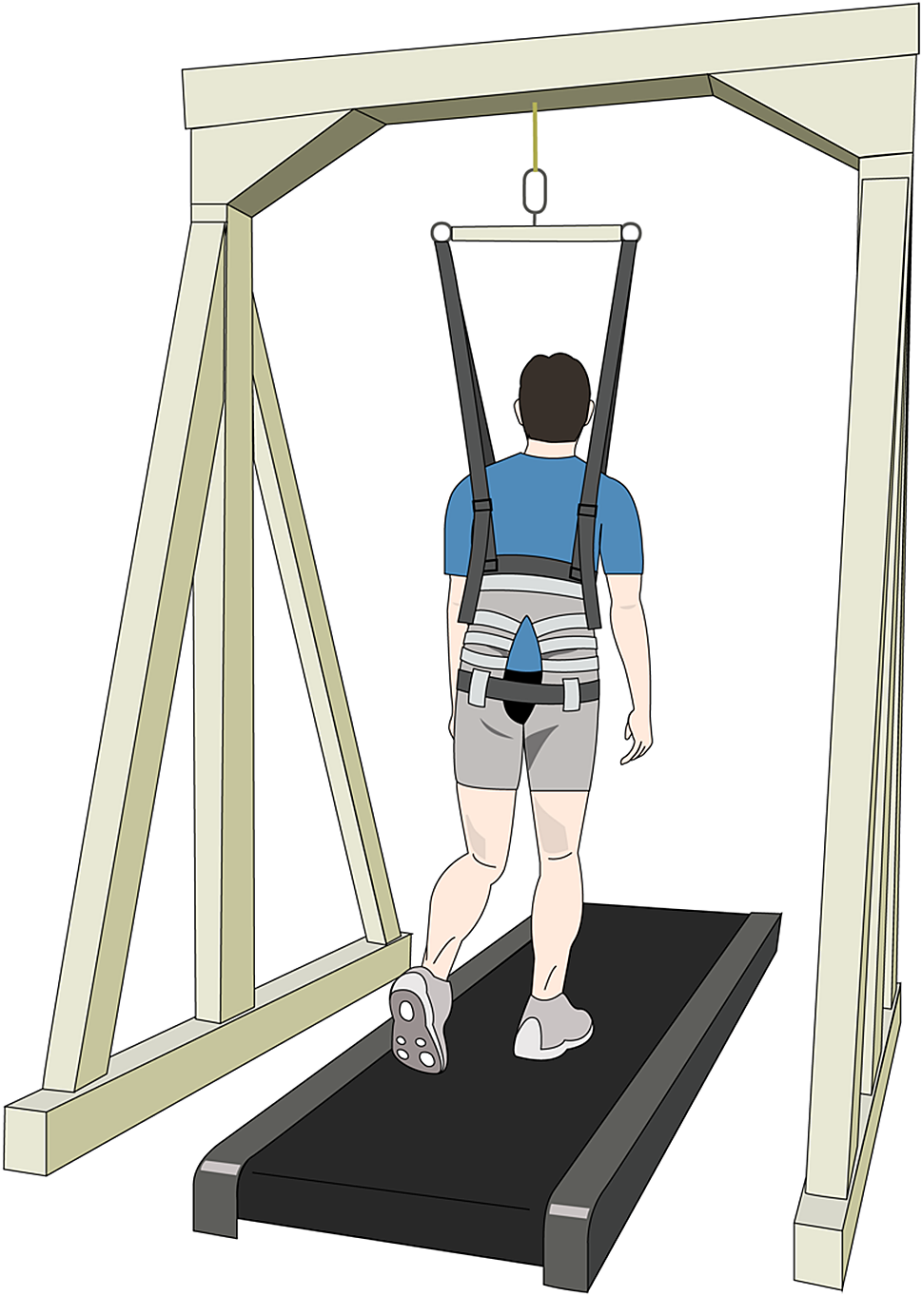
Reduced gravity simulator during walking. Participants walked on a treadmill while a body weight support system vertically pulled up on the participants’ torsos using a harness attached to their thighs and waist to reduce the load on supporting legs corresponding to 0, 40, 62, 84, or 93% of their body weights. This figure was created by one of the authors of this paper, Shota Hagio, using Adobe Illustrator 2020 (Adobe Inc., San Jose, CA, USA; https://www.adobe.com/jp/products/illustrator.html).

Therefore, we seek to reveal the gravity-dependent modulation of muscle activation patterns in walking regarding both spatial and temporal modules. Walking under 1 g of gravity was simulated using a vertical BWS system (Fig. 1). First, we needed to test whether individual spatial and temporal modules were shared among the different gravity levels. To this end, spatial modules and temporal modules in each participant and gravity conditions were extracted from the EMGs using nonnegative matrix factorization^24,33^. The representative modules that resulted from classifying the modules in each participant into several clusters were then compared among different gravity levels. Second, how pairs of spatial and temporal modules are modulated should be tested depending on the gravity levels. Tensor decomposition techniques make it possible to unravel this question by extracting task-dependent variables that vary in gravity, walking speed and participants in addition to fixed spatial and temporal modules^34,35^. The present results will provide a better understanding of neuromuscular control during walking under hypogravity conditions.

## Results

### Muscle activity

**Figure 2:**
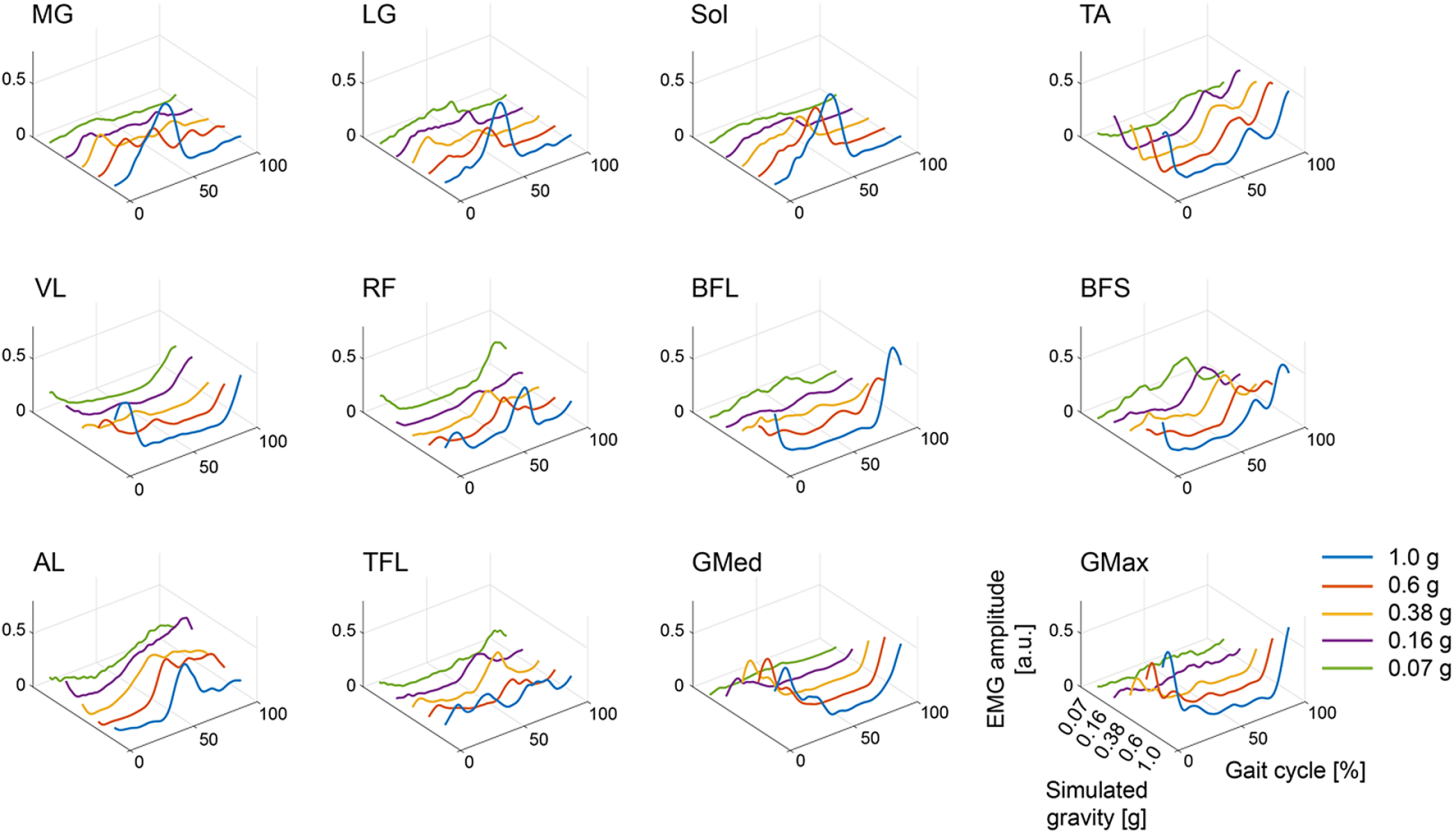
Muscle activity across each gravity. Rectified and filtered electromyographic (EMG) activity normalized to the peak activity in a gravity condition of 1 g are shown. The activation profiles for the individual gravity levels (1 g, 0.6 g, 0.38 g, 0.16 g, and 0.07 g) are distinguished by colour. Each data point is an average across all participants and 10 gait cycles (from the onset of the right leg to the next; normalized to 200 time bins) at a walking speed corresponding to the Froude number (*Fr*) ~ 0.25. The abbreviations of the muscles are as follows: medial head of the gastrocnemius muscle (MG), lateral head of the gastrocnemius muscle (LG), soleus (SOL), tibialis anterior (TA), vastus intermedius (VL), rectus femoris (RF), biceps femoris (long head, BFL), biceps femoris (short head, BFS), adductor longus (AL), tensor fasciae latae (TFL), gluteus medius (GMed), and gluteus maximus (GMax).

The amplitude of the EMGs in the plantar flexor muscles, including the medial head of the gastrocnemius muscle (MG), lateral head of the gastrocnemius muscle (LG) and soleus (Sol), hip abductor, gluteus medius (GMed), and the extensor muscle, and gluteus maximus (GMax), decreased with decreasing simulated gravity at a Froude number (*Fr*) of ~ 0.25 (Table 1), whereas the temporal profiles of the EMGs were similar among the different gravity levels (Fig. 2). In a dorsiflexor muscle and the tibialis anterior (TA), the spatiotemporal EMG characteristics were similar from the gravity conditions of 1 g to 0.16 g but changed at 0.07 g. Although the temporal features in the muscle activity described above were approximately maintained despite the changing gravity levels, the EMGs in the other muscles located in the thigh demonstrated complicated changes in temporal features in addition to their amplitudes.

**Table 1:** Treadmill speed determined by the Froude number (*Fr*) corresponding to 0.25.

Gravity level	1 g	0.6 g	0.38 g	0.16 g	0.07 g
Mean (km/h)	5.05	3.91	3.11	2.02	1.34
SD (km/h)	0.11	0.09	0.07	0.05	0.03

**Figure 3:**
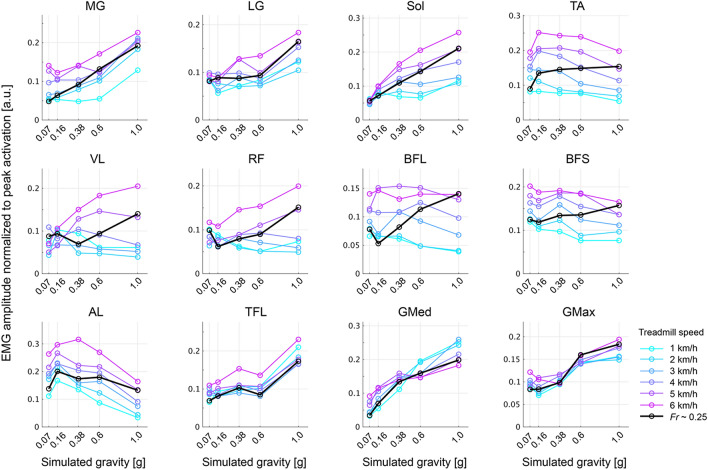
Mean muscle activity over time for all gravity levels and treadmill speeds. The processed EMGs shown in Fig. 2 are averaged across a series of gait cycles. The circles and lines for the individual treadmill speeds (1–6 km/h and speed corresponding to *Fr* ~ 0.25) are distinguished by colour.

In general, the amplitude of EMG activity increased with increasing walking speed in the reduced gravity conditions as well as the gravity condition of 1 g (Fig. 3). However, in the quadriceps muscles, the vastus lateralis (VL) and rectus femoris (RF), this relationship was inverted at lower gravity levels, i.e., 0.16 and 0.07 g. In contrast, increasing walking speed led to less recruitment of the GMed, and the relationship was also reversed under a gravity condition of 0.38 g. The EMG activity of the biceps femoris (long head, BFL) decreased with decreasing gravity at *Fr* ~ 0.25, while this modulation depended on walking speed rather than the change in gravity levels. Importantly, some walking speeds required monotonic modulation of the EMG amplitude depending on the gravity levels, but in others, the EMG amplitude was modulated as a U-shaped or inverted U-shaped function of the gravity. For example, the inverted U-shaped function of the adductor longus (AL) peaked at 0.38 g and 0.16 g with walking speeds of 6 km/h and under 5 km/h, respectively. Furthermore, the EMG amplitude of the TA monotonically increased with decreasing gravity at a walking speed under 3 km/h, but above 4 km/h, it reached a peak value at a gravity of 0.16 g. These results indicate that the interaction between the gravity level and the walking speed changed lower limb muscle activities and that the gravity- and speed-dependent modulation patterns varied in individual muscles.

### Selection of the number of spatial and temporal modules

**Figure 4:**
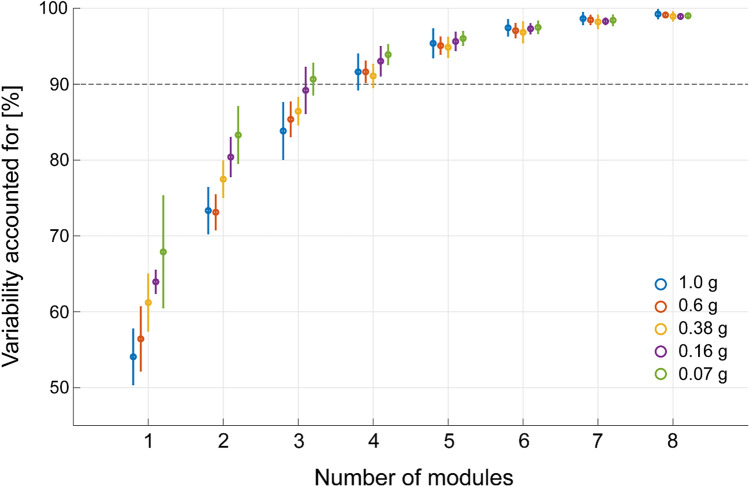
Goodness of fit of the EMG data reconstruction. The variability of the original EMG data matrix accounted for by 1–8 modules is shown. The circles and lines for the individual gravity levels are distinguished by colour. Each data point and bar are an average ± standard deviation across participants at a speed corresponding to *Fr* ~ 0.25.

Spatial and temporal modules were extracted from the complex EMGs to compare the low-dimensional representation of multiple muscle activities among the gravity conditions during walking at *Fr* ~ 0.25. We needed to test whether the numbers of modules changed among the gravity levels by comparing the variability of EMG data accounted for (VAF) by the reconstructed data across conditions (Fig. 4). Significant main effects of the number of modules (*F*(11, 88) = 2082.0, *p* < 0.001, partial *η*^2^ = 1.00) and conditions (*F*(4, 32) = 12.38, *p* < 0.001 partial *η*^2^ = 0.61) and a significant interaction between the number of modules and the conditions (*F*(44, 352) = 12.52, *p* < 0.001, partial *η*^2^ = 0.61) were observed in the VAF value. Accordingly, the fewest number of modules was determined to be 4 in the simulated gravity conditions from 1 to 0.16 g and 3 in the gravity condition of 0.07 g, and it could explain more than 90% of the total variance in the original EMG signals. Post hoc pairwise comparisons also revealed that the VAF value was higher in the gravity condition of 0.07 g than in the gravity conditions from 1 to 0.16 g if the number of modules was 3 (*p* < 0.05).

### Spatial modules

**Figure 5:**
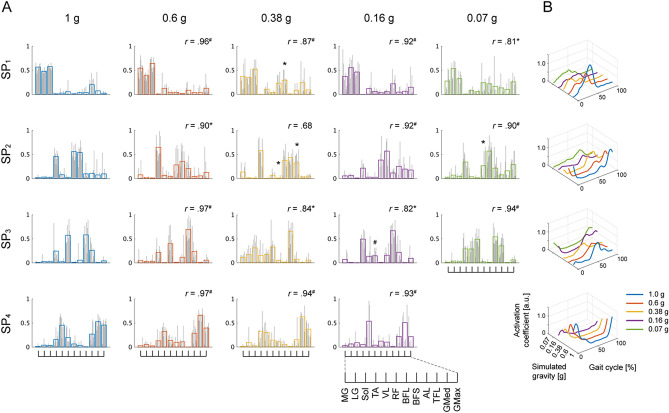
Modules clustered based on the spatial modules. Modules extracted from the EMG data matrix at *Fr* ~ 0.25 are shown. (**A**) The bar graphs represent weighting vectors for each muscle, i.e., a spatial module. Spatial modules identified from all participants were classified into a set of 4 clusters in each gravity level. The grey individual bar graph indicates a spatial module identified in each participant, and the coloured solid bar graph indicates the average over participants. (**B**) The line plots indicate the amplitude of the activation coefficient, i.e., a temporal module, averaged across participants. The different columns of the spatial modules and different colours of the temporal modules denote the 5 different gravity levels. The spatial modules corresponding to the same cluster, i.e., SP_1_ to SP_4_, and the corresponding temporal modules are arranged in the same row. The similarity of the spatial modules between the gravity condition of 1 g and each of the other gravity levels in each cluster (SP_1-4_) is shown as the cosine similarity (*r*). Symbols (*, #) indicate a significant difference in the weighting vectors of all or each muscle; *p < 0.05 and ^#^*p* < 0.01.

The spatial modules extracted during walking at *Fr* ~ 0.25 were classified into 4 clusters across participants in each gravity condition (Fig. 5). The number of cluster modules was determined according to the largest mean silhouette value (see “Methods”; Table 2). The similarity analysis after further classification among gravity conditions demonstrated that the cosine similarity of almost all pairs of the spatial modules (SP_1-4_) between the gravity condition of 1 g and each of the remaining gravity levels was above chance level (see *r* value in Fig. 5), indicating that the underlying structure of the spatial modules was shared among each gravity level. The spatial module SP_1_ consisted of the plantar flexor muscles, including the MG, LG and Sol, which activated approximately 40% of a gait cycle corresponding to the propulsive phase. The statistical analysis for testing the differences in muscle weights of SP_1_ revealed significant main effects of muscles and gravity conditions and a significant interaction between the muscles and the conditions (SP_1_ in Table S1). The post hoc analysis for testing the difference in muscle weights between the gravity condition of 1 g and the remaining gravity conditions demonstrated larger weights of the biceps femoris (short head, BFS) in the gravity condition of 0.38 g (*p* = 0.027). Module SP_2_ was constructed by a dorsiflexor muscle, the TA, and knee flexion muscles, the BFL and BFS, which contributed to the last swing phase. Significant main effects of muscles and a significant interaction between the muscles and the conditions were observed, whereas no effect was found in the gravity conditions (SP_2_ in Table S1). The post hoc tests revealed differences in the muscle weights of the BFL between the gravity conditions of 1 g and 0.38 g and between the gravity conditions of 1 g and 0.07 g and in the muscle weights of the tensor fasciae latae (TFL) between the gravity conditions of 1 g and 0.38 g. Moreover, the cosine similarity between the pair of spatial modules in the gravity conditions of 1 g and 0.38 g was lower than the chance level (*r* = 0.68). Module SP_3_, constructed by the TA; a knee extensor and hip flexor muscle, the RF; and an adductor muscle, the AL, was recruited early in the swing phase. While significant main effects of muscles and a significant interaction between the muscles and the conditions were observed, no significant effect of the gravity conditions was found (SP_3_ in Table S1). A knee extensor muscle, the VL, and muscles spanning the hip joint, the TFL, GMed and GMax, composed module SP_4_, which was activated in the initial contact phase. Although significant main effects of the muscles and a significant interaction between the muscles and the conditions were observed, no significant effect of the gravity conditions was found (SP_4_ in Table S1). The post hoc tests showed that there was no significant difference in muscle weights between the gravity conditions of 1 g and the other gravity conditions.

**Table 2:** Silhouette value in each number of clusters.

#Clusters	2–3	4	5	6	7	8	9	10
spatial module	0	0.73^a^	0.64	0.57	0.53	0.51	0.50	0.51
temporal module	0	0.84^a^	0.81	0.76	0.73	0.71	0.70	0.70

The number of clusters for the spatial and temporal modules was defined as 4 based on the largest silhouette value.

^a^Indicates the optimal number of clusters.

Moreover, the overall similarity of modules between the gravity conditions of 1 g and each of the remaining gravity levels was quantified as the median value of the cosine similarity for all pairs of modules that were matched to each other. The median cosine similarity between pairs of spatial modules was above a chance level in the gravity condition of 0.6 g but below a chance level in the simulated gravity conditions from 0.38 to 0.07 g (Fig. 7A). Moreover, a significant main effect of the pairs of gravity conditions on the cosine similarity value was observed (*χ*^2^ = 15.10, *p* = 0.0017). The value of the cosine similarity was smaller below the gravity condition of 0.38 g than the value between the gravity conditions of 1 g and 0.6 g. These results indicate that the spatial modules extracted in each gravity condition were classified into 4 clusters whereas the individual muscle weights of the spatial modules in the gravity conditions of 1 g was partially changed as decreasing the gravity levels below 0.38 g.

### Temporal modules

**Figure 6:**
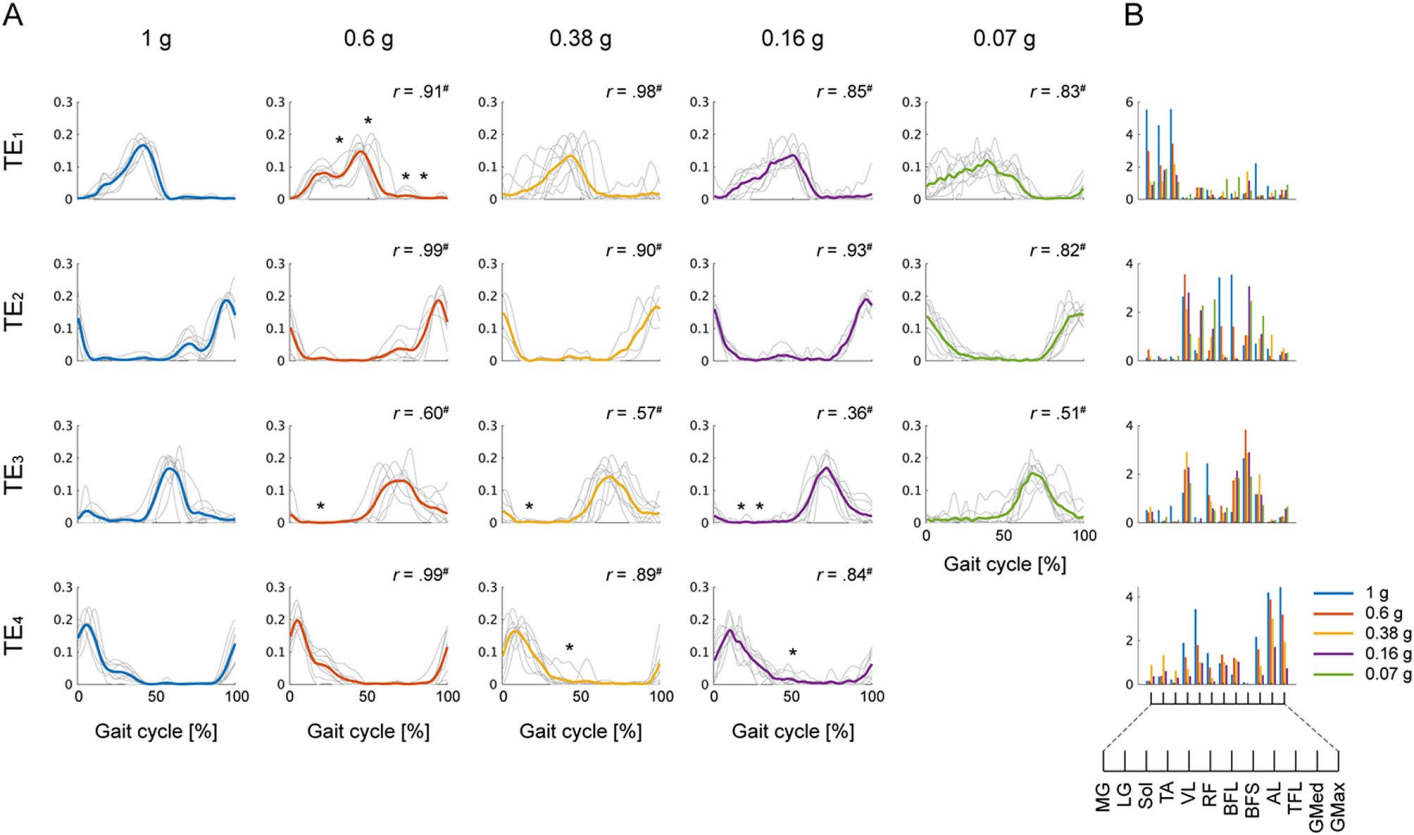
Modules clustered based on the temporal modules. Modules extracted from the EMG data matrix at *Fr* ~ 0.25 are shown. (**A**) The line plots represent a temporal module. Temporal modules identified from all participants were classified into a set of 4 clusters in each gravity level. The grey individual line indicates a temporal module identified in each participant, and the coloured solid line indicates the average. (**B**) The bar graphs indicate spatial modules averaged across participants. The different columns of the temporal modules and different colours of the spatial modules represent the 5 different gravity levels. The temporal modules corresponding to the same cluster, i.e., TE_1_ to TE_4_, and the corresponding spatial modules are arranged in the same row. The similarity of the temporal modules between the gravity condition of 1 g and each of the other gravity levels in each cluster (TE_1-4_) is shown as the correlation coefficient (*r*). Symbols (*, #) indicate a significant difference in the activation coefficients of all or each phase; *p < 0.05 and ^#^*p* < 0.01.

**Figure 7:**
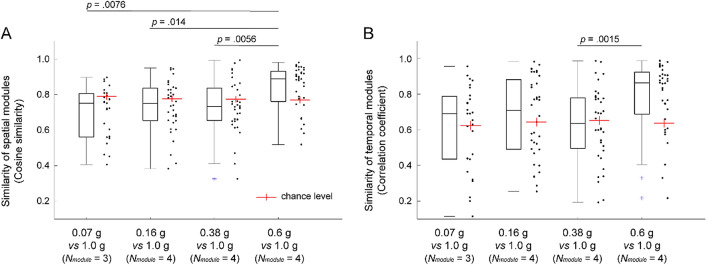
Structural change in the modules. The similarity of the spatial (**A**) and temporal (**B**) modules between the gravity condition of 1 g and each of the remaining gravity levels was calculated across all pairs of modules that were matched to each other by maximizing the cosine similarity between each spatial module pair and the correlation coefficient between each temporal module pair. Each dot indicates the similarity of each module pair, and the boxplot represents their distribution. The red crossed line indicates the chance level.

The temporal modules were classified into 4 clusters across participants in each gravity condition corresponding to the classification observed in the spatial modules (Fig. 6, Table 2). The activation profiles of temporal modules were similar among each gravity level (see *r* value in Fig. 6), indicating that the underlying temporal profile of the modules was preserved among each gravity level. However, in modules TE_1_, TE_3_ and TE_4_, significant main effects involving gravity levels were observed depending on the walking phase (Table S2). In module TE_2_ contributing to the late swing phase for walking, no significant effect of the difference in the gravity levels was found (TE_2_ in Table S2). The change in the correlation coefficient between pairs of temporal modules was similar to that observed in the spatial modules; the median correlation coefficient was above a chance level in the gravity condition of 0.6 g and equivalent to a chance level in the simulated gravity conditions from 0.38 to 0.07 g (Fig. 7B).

### Gravity-dependent modulation of spatial and temporal modules

The reduced gravity partially changed the spatial and temporal modules, while how these modules were modulated in a gravity-dependent manner remains unclear. The results described above also showed that the underlying structure of the spatial and temporal modules was shared among each gravity level that was classified into each of 4 clusters (Figs. 5 and 6). Accordingly, the subsequent analysis was performed from the perspective of how the shared spatial and temporal modules were modulated depending on the reduced gravity condition.

The tensor decomposition technique enabled us to estimate the gravity-dependent variables in addition to fixed spatial and temporal modules (ST modules). The number of extracted modules was defined as that corresponding to the number of clusters, i.e., 4, which was determined in the extraction of spatial and temporal modules in each gravity level (Figs. 5 and 6). The 4 modules could explain 47.7% of the total variance in the original EMG signals. Despite the low data reconstruction relative to that in previous studies, the spatial and temporal modules extracted at each gravity level could be reconstructed by the combination of the spatial and temporal components of the ST modules (Figs. S1 and S2). Here, we focused on the gravity-dependent variables in each ST module. The ANOVA results for the effect of gravity levels on the task-dependent variables in each ST module are shown in Table 3. The post-hoc power analysis revealed a calculated power > 0.99 for the ST modules.

**Table 3:** One-way repeated-measures ANOVA tables for the effect of gravity levels on the task-dependent variables, ***t***, of ST modules.

	*df*	*F*	*p*	Partial *η*^2^
ST_1_	4, 32	2.94	0.036	0.27
ST_2_	4, 32	29.32	< 0.001	0.79
ST_3_	4, 32	47.37	< 0.001	0.86
ST_4_	4, 32	4.07	0.0088	0.34

The task-dependent variable in ST_1_ involved with SP_2_, SP_3_, TE_2_ and TE_3_ appeared to be constant throughout all the gravity levels. A significant main effect of gravity levels was observed whereas the post hoc pairwise comparisons did not show a difference in the task-dependent variables estimated in each pair of gravity levels. However, the other ST modules demonstrated the modulation of gravity-dependent variables. In ST_2_ related to SP_1_ and TE_1_, the task-dependent variables monotonically decreased below 0.6 g compared with the gravity condition of 1 g. In ST_3_ involved with SP_4_ and TE_3_ or TE_4_, the task-dependent variables were equivalent between 1 and 0.6 g but monotonically decreased below 0.38 g. Importantly, the disappearance of SP_4_ and TE_4_ in the gravity condition of 0.07 g that was observed in the results of matrix factorization (Figs. 6 and 7) was represented as the task-dependent variable of ST_3_ close to 0, indicating that the different decomposing techniques could extract the common characteristics of gravity-dependent modulation of the spatial and temporal modules. Surprisingly, the task-dependent variables in ST_4_ relevant to SP_3_ and SP_4_ and TE_2_ or TE_4_ were modulated as a U-shaped function of gravity; the variables decreased in gravity conditions of 0.6 and 0.38 g but were comparable under gravity conditions of 0.16 and 0.07 g compared with the gravity condition of 1 g. These results indicate that spatial and temporal coordination of multiple muscle activities was modulated in a module-dependent manner as a function of gravity levels.

### Gravity-dependent modulation of modules at different walking speeds

**Figure 8:**
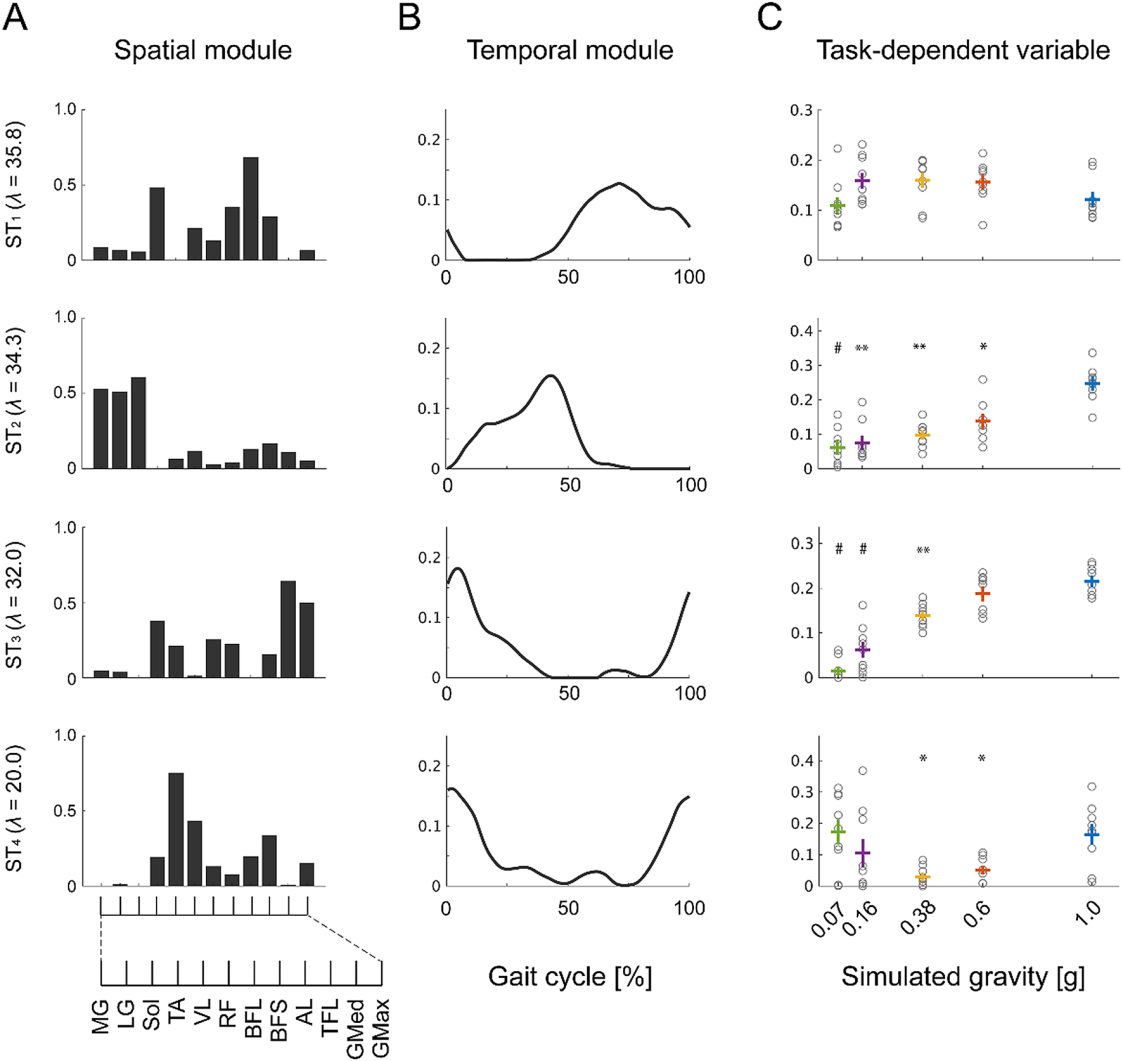
Spatial and temporal modules and task-dependent variables. Spatial (**A**) and temporal (**B**) modules and corresponding task-dependent variables (**C**) extracted from the EMG data tensor at *Fr* ~ 0.25 are shown. *λ* denotes the scaling factor. Each circle of the task-dependent variables indicates the data for individual participants. The mean and standard error of the means are represented as horizontal and vertical lines of a coloured crossed line in each gravity level. Symbols (*, #) indicate a significant difference in the task-dependent variables between a gravity condition of 1 g and the other gravity levels; *p < 0.05, **p < 0.01 and ^#^*p* < 0.001.

**Figure 9:**
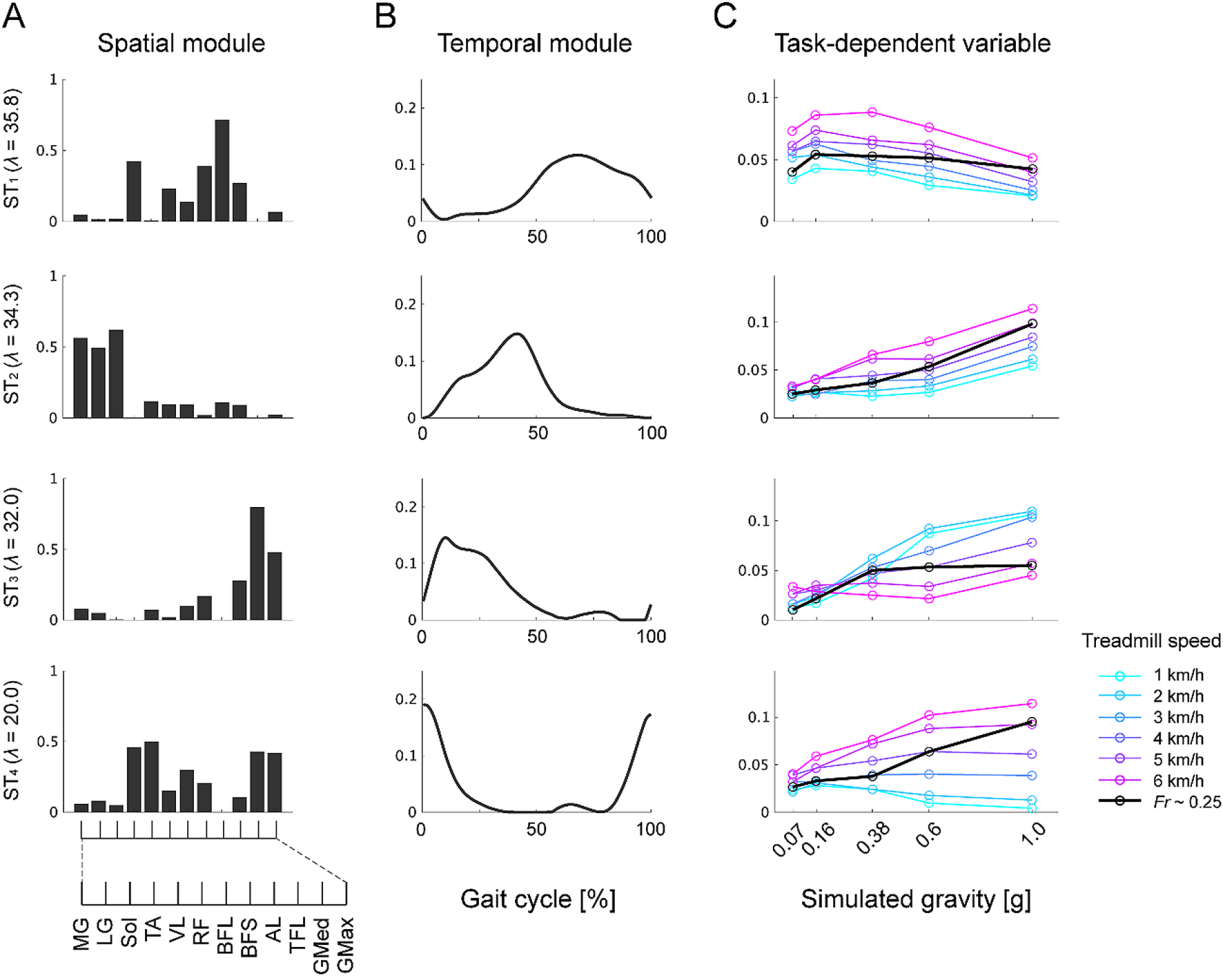
Spatial and temporal modules and task-dependent variables across walking speeds. Spatial (**A**) and temporal (**B**) modules and corresponding task-dependent variables (**C**) extracted from the EMG data tensor at 7 walking speeds (1–6 km/h and a speed corresponding to *Fr* ~ 0.25) are shown. *λ* denotes the scaling factor. The circles and lines for the task-dependent variables in the individual treadmill speed are distinguished by colour.

We also investigated the gravity-dependent modulation of ST modules at different treadmill speeds (Fig. 9). Notably, the ST modules shown in Figs. 8 and 9 did not completely correspond to each other because of the different composition of the EMG tensor for the tensor decomposition (see “Methods”), but the spatial structure and the temporal profile were similar to each other. The behaviour of modulation of task-dependent variables in ST_1_ and ST_2_ was similar to the different treadmill speeds. However, the modulation profiles in ST_3_ and ST_4_ were different among the treadmill speeds. In ST_3_, the task-dependent variables monotonically decreased from 1 to 0.07 g at speeds below 4 km/h but were approximately constant at speeds above 5 km/h. In ST_4_, the variables monotonically decreased from 1 to 0.07 g at speeds above 5 km/h, whereas a monotonic increase in the variables was observed at speeds below 2 km/h. Therefore, the interaction between the gravity level and walking speed could determine the modulation of spatial and temporal coordination of multiple muscle activities.

## Discussion

This study investigated how the spatial and temporal modules for the activity of multiple muscles during walking were modulated depending on gravity. All of the spatial and temporal modules extracted from the EMG data matrix in each gravity level were classified into 4 clusters shared among the different gravity levels whereas individual muscle weights and the time profiles of activation coefficients in a gait cycle were partially changed due to the reduced gravity compared with those under the gravity condition of 1 g. Moreover, the gravity-dependent variables estimated using EMG tensor decomposition revealed that some sets of spatial and temporal modules were modulated in a monotonical manner, and the other was recruited in a nonlinear U-shaped fashion as a function of gravity. The results also demonstrated that the form of the gravity-dependent modulation of modules systematically changed depending on the walking speed.

The pendulum-like mechanism of walking is mechanically impeded as gravity decreases^10,11^, resulting in a change in the desired muscle activity^6,18,19,36^. Indeed, consistent with previous studies, the amplitude of muscle activities was modulated depending on gravity levels, and behaviours were different among individual muscles (Figs. 2 and 3)^6,19^. However, the changes in muscle activity as a function of gravity levels could be explained by the combination of fixed spatial modules comparable with those reported in previous literature (Fig. 5)^25,31,37,38^. Previous physiological and simulation studies have reported that a wide range of locomotor-like movements, such as walking, running and obstacle clearance, can be captured by the combination of a few shared spatial modules with task-specific modules^25,39,40^, indicating that the modules represent functional units as locomotor primitives^26,37,41,42^. The shared structure of the spatial modules among different gravity conditions would reflect the robustness of the locomotor primitives against the mechanical effects of low gravity on the locomotor system.

On the other hand, the individual muscle weights partially changed below the gravity condition of 0.38 g; however, the systematic relationship to gravity was weak (Figs. 5 and 7). This partial modulation of the spatial modules was involved with the altered contribution of the spinal circuit depending on the gravitational load^7,16^. A previous study demonstrated that after deafferentation, most deafferented spatial modules were consistent with the intact modules, whereas individual muscle weights of several spatial modules were partially altered during swimming and jumping movements of bullfrogs^43^. In the present study, the altered loading of the lower limbs might change the responses of individual muscles to load-related mechanoreceptor stimuli and subsequently lead to modulation of the individual muscle weights.

In addition to the spatial modules, the temporal modules were shared at each gravity level (Fig. 6). The invariant profiles of temporal modules were previously observed during locomotion with voluntary tasks and a BWS condition, implying locomotor programs that determine basic activation timing of muscles^28,29,31^. However, the profiles of several temporal modules in some phases were partially variable among gravity levels (Figs. 6 and 7). The partial changes in the temporal modules reflect the effect of gravity on sensory flow which modulates the activation coefficients of the modules rather than the muscle weights^43^. Acute changes in activation coefficients rather than muscle weights were also observed in a previous parabolic flight study regarding the control of upright posture during short-term exposure to microgravity^44^.

The task-dependent variables estimated using a tensor decomposition demonstrated the gravity-dependent modulation of both spatial and temporal modules (Fig. 8). With decreasing gravity, the task-dependent variables in the ST_2_ module monotonically decreased, consistent with an antigravity function of the relevant muscles, i.e., the plantar flexors^6,19^. This module was activated in the propulsive (ST_2_) phase to produce mass acceleration of all body segments that are represented in ground contact forces^45^. Thus, the decremental pattern of the task-dependent variables corresponds to a linear reduction in the peak of the ground contact force with decreasing simulated gravity^19^.

The peak activation timings of modules ST_3_ and ST_4_ corresponded to the moment of foot contact; therefore, these modules would begin to act prior to foot contact (Fig. 8)^46^. Gravity-dependent modulation of the lower limb muscle preactivation has been previously reported in locomotor-like tasks, such as bouncing and drop landing movements^47,48^. The reduced gravity monotonically decreased the task-dependent variables in the ST_3_ module and decreased the muscle activity observed in the aforementioned studies, while the task-dependent variables in the ST_4_ module, which consisted of the knee extensors, were modulated in a U-shaped fashion as a function of gravity. With body weight unloading, EMG activity in the knee extensors paradoxically increased, and this could not be predicted simply by the static load during stance^19^. The pre-activation of the module rather than the response to load-related sensory feedback should be adjusted by modifying the internal representation of walking movement based on the interaction between the body and the gravitational environment. The gravity-dependent nonlinear modulation of the module might reflect a higher level contribution of locomotor control.

The vertical BWS system is one of the more commonly used systems to simulate reduced gravity locomotion^19,49^. However, the limitation of the simulator is that the load on the supporting limbs can be reduced, while the swinging limb experiences 1 g^50^. Indeed, although gravity-dependent modulation was observed only in the ST_2-4_ modules, relevant to the supporting phase during walking, definite differences in the task-dependent variables of the ST_1_ module involved with the swinging phase were not observed among the gravity levels (Fig. 8). However, the significant effect of the gravity conditions on the task-dependent variables of ST_1_, indicating that the unloading during the supporting phase affected the modulation of muscle activities during the swinging phase, cannot be overlooked. Simulators close to real reduced gravity conditions are needed for a comprehensive understanding of the gravity-dependent modulation of the modules, including the features in the swinging phase^8,9,50^.

Thus far, we have discussed the gravity-dependent modulation of the modules at a roughly optimal walking speed corresponding to *Fr* ~ 0.25 in which the dynamic state of the body was geometrically similar among each gravity level because of the pendulum-like behaviour of the limbs^51,52^. However, not only gravitational load but also walking speed affect the generation of muscle activation patterns and their regulation based on modules^19,34,42,53^. As reported in previous studies, muscle activity at different walking speeds could be represented by spatial and temporal modules comparable with those at each walking speed^25,30,42^, in which task-dependent variables were systematically changed as a function of both gravity and treadmill speed in a related phase-dependent manner (Fig. 9). With reduced gravity, the task-dependent variables in the ST_3_ module recruited just after foot contact linearly decreased, especially at slower walking speeds. Because the net load due to gravity has a great effect on the lower limbs early in the supporting phase, as observed by the correlation between the first peak of the ground reaction force and a gravity level^19^, the characteristics of the modulation of the task-dependent variables, i.e., the linear decrease with reduced gravity and a higher value at lower walking speeds, can be explained by the response to the dynamic principle because of the inverted pendulum-like behaviour of the limbs as follows: $\left[net load\right] \sim m\left(g-\frac{{v}^{2}}{L}\right)$, where *m* is the body mass, *L* is the characteristic leg length, *g* is the acceleration of gravity, and *v* is the walking speed^51^. As the walking speed increased, however, the module recruitment pattern was altered compared with the slower walking speeds, presumably because the dynamic principle was broken, i.e., $\frac{{v}^{2}}{gL}>1$. In the ST_2_ module acting in the acceleration phase for forward propulsion of body mass, linear modulation of the task-dependent variables was observed depending on the gravity levels as well as the case of the ST_3_ module, whereas the value of the task-dependent variables was higher at faster walking speeds. During walking, mechanical energy is largely conserved by the conversion of kinetic energy for forward acceleration of body mass into gravitational potential energy^54^. The pattern of module modulation in ST_2_ might be related to mechanical energy consumption, which decreases with decreasing gravity and walking speed^14,55^. In the ST_4_ module, while load-dependent modulation of the task-dependent variables was observed at higher walking speeds, the pattern at slower walking speeds was systematically shifted to an incremental pattern with decreasing gravity. This paradoxical modulation was previously reported regarding the quadriceps muscles and their relevant module as well as the ST_4_ module, which may reflect different biomechanical demands on postural stability and the altered dynamics of the lower limb movements between higher and slower walking speeds^19,53^. Future studies that use dynamic simulation of walking, including the modularity of multiple muscle activities in various gravitational environments and walking speeds, will help improve the understanding of the modulation of the modules that are involved with locomotor dynamics in relation to gravity and walking speed^39,55^.

In the present study, the spatial and temporal modules were first extracted using matrix factorization (Figs. 5 and 6); then, the task-dependent variables under the assumption of fixed spatial and temporal modules were estimated using tensor decomposition (Fig. 8). The two different types of analysis are needed not only to elucidate the effect of gravity on locomotor control in different dimensions but also to test a supposition for tensor decomposition. The several models were advocated that explained modularity in both spatial and temporal muscle activation patterns, such as a time-varying modularity model^22,56^ and a space-by-time modularity model^57^. However, the shared underlying structure of both the spatial and temporal modules among the gravity conditions that was identified with the matrix factorization were corresponding to the basic assumption of the tensor decomposition (CP) model. Indeed, the spatial and temporal modules extracted in the two different types of techniques were related to each other (Figs. S1 and S2), as seen in a previous locomotion study^34^. Furthermore, the unweighted SP_4_ and TE_4_ modules in a gravity condition of 0.07 g were represented by the task-dependent variable close to 0 in the ST_3_ module that was involved with SP_4_ and TE_4_. These results indicate that the gravity-dependent modulation of the modules was demonstrated as the task-dependent variable while maintaining the features of the extracted modules using matrix factorization. However, the percentage of EMG data variance accounted for by the ST modules yielded a low value. This was because the limitation of the ST modules that reflected generic features of muscle activities was shared among all participants and gravity levels but could not explain the inter-subject and inter-gravity variabilities of the modular structures, as demonstrated in the variability of the spatial and temporal modules extracted using NMF.

Extraterrestrial space development, such as on the moon or Mars, has been challenged by the human race. In unexperienced gravitational environments, humans must maintain the dynamic stability of locomotion by rapidly recalibrating the gravity-dependent sensorimotor transformation^4,5^. Failure of locomotor control on extraterrestrial planets can cause a fall that may be life threatening^1^. Therefore, understanding how locomotor control is corrected against sudden transitions between different gravity states is crucial. In this study, we simulated extraterrestrial walking, such as on Earth, Mars, the moon, and Pluto, and gained a systematic understanding that locomotor stability can be maintained by regulating motor output based on robust spatial and temporal modules depending on hypogravity levels. Moreover, partial BWS gait therapy is the most common medical practice to facilitate regaining the interaction between body mechanics and gravitational forces impaired by neurological insults such as spinal cord injury and stroke^58–60^. Furthermore, our results provide a perspective about the gravity-dependent modulation of locomotor muscle activity not only in the field of space science but also in clinical rehabilitation.

## Conclusion

The simulated reduced gravity changed the amplitude of lower limb muscle activities during treadmill walking. However, the locomotor modules coordinating multiple muscle activation patterns are shared among different hypogravity conditions despite partial changes being observed in individual muscle weights and phase-dependent activation coefficients. The contribution of the prescribed spatial and temporal modules was systematically modulated as a function of gravity and walking speed. We speculate that the gravity-dependent modulation of the locomotor modules can be controlled by higher locomotor centres based on load-related sensory feedback and biomechanical demands according to movement dynamics. We provided a systematic understanding of the regulation of module-based muscle activity to maintain walking stability in various hypogravity conditions.

## Methods

### Participants

Nine healthy male subjects participated in this study. The age, height, and body mass of the participants (mean ± standard deviation) were 24.2 ± 3.0 years, 172.2 ± 7.8 cm, and 64.4 ± 6.7 kg, respectively. They gave their informed consent for the study after receiving a detailed description of the purpose, potential benefit, and risks during the experiment. All procedures used in this study were in accordance with the Declaration of Helsinki and were approved by the Committee for Human Experimentation at the Graduate School of Human and Environmental Studies, Kyoto University (approved number: 29-H-16).

### Experimental setup

The participants were asked to walk on a motorized treadmill (T652 Treadmill, SportsArt co., Tainan city, Taiwan). The running surface of the treadmill was 1.53 m long and 0.55 m wide. The participants were instructed to fixate on a visual target placed in front of them at eye level. The treadmill speed was maintained at seven different speeds: 1, 2, 3, 4, 5 and 6 km h^−1^ and a subject-specific speed determined by the Froude number (*Fr*) corresponding to 0.25 based on the leg length and the simulated gravity levels (Table 1)^52^. During walking, the BWS system vertically pulled up on the participants’ torsos using a harness attached to their thighs and waist to reduce the load on the supporting legs. The rope pulling up their bodies was connected to weights corresponding to 0, 62%, 84%, 93% or 40% of their body weights. These BWS conditions simulated walking with 5 kinds of gravity conditions: 1 g, 0.38 g, 0.16 g, and 0.07 g, corresponding to gravity on Earth, Mars, the moon, and Pluto, respectively, and 0.6 g. Therefore, the participants underwent a total of 35 walking trials: 7 treadmill speeds × 5 gravity levels. All participants initially walked with a gravity of 1 g, and then the order of the other simulated gravity levels was randomized across participants. In each simulated gravity level, the trials at 7 different treadmill speeds were randomly assigned over 40 cycles. Between each gravity condition, the participants walked for 3 min with a gravity of 1 g at *Fr* ~ 0.25. The participants familiarized their walking with each simulated gravity level so as not to run or jump up when the gravity level was switched and so that they could perform stable walking after a few minutes^19^.

### Data recording

During walking, the positions of reflective markers attached at the greater trochanters (GT) and lateral malleolus (LM) on the right leg were recorded at 100 Hz using a motion capturing system (OptiTrack V100, NaturalPoint Inc., Oregon, United States of the America). Surface electromyograms (EMGs) were recorded from 12 muscles on the right leg: the medial head of the gastrocnemius muscle (MG), lateral head of the gastrocnemius muscle (LG), soleus (SOL), tibialis anterior (TA), vastus lateralis (VL), rectus femoris (RF), biceps femoris (long head, BFL), biceps femoris (short head, BFS), adductor longus (AL), tensor fasciae latae (TFL), gluteus medius (GMed), and gluteus maximus (GMax). The EMGs were recorded using bipolar Ag–AgCl electrodes. Each electrode had a diameter of 10 mm, and the interelectrode distance was 20 mm. The EMG electrodes were placed according to the recommendations from SENIAM (seniam.org). The skin was prepared by shaving the hair, surface abrasion and alcohol cleansing. Reference electrodes were placed on the LM. Each electrode was taped on the muscle belly and connected to an amplifier (SX230, Biometrics Ltd., Newport, United Kingdom), where the EMG signal was bandpass filtered (5–480 Hz) and amplified (total gain, 1000). All electrical signals were recorded and stored at a sampling frequency of 1,000 Hz on the hard disk of a personal computer using a 16-bit analogue-to-digital converter (PowerLab/16SP; AD Instruments, Sydney, Australia).

### Data processing

The limb axis was defined as GT-LM. The moment of foot contact was determined as the time of maximum elevation of the limb axis^19,61^. The gait cycle was defined as the time between foot contact and the next ipsilateral foot contact in the right leg. The raw EMG signals were high-pass filtered at 100 Hz using a zero phase-lag fourth-order Butterworth filter, demeaned, rectified, and low-pass filtered at 10 Hz using custom MATLAB routines. We used the relatively aggressive high-pass filter (100 Hz) because such high-pass filtering improves the information density of the movement-related signals, which captures a better relationship between neuromuscular activity and mechanical variables, such as muscle force and torque, than conventional filtering^25,62,63^. The EMG traces were time-interpolated over individual gait cycles to fit a normalized 200-point time bins^25^ and then averaged across 10 gait cycles only for the analysis of ensemble data in each participant, gravity level and treadmill speed^27^. We subtracted the minimum over the cycle from each EMG trace and normalized the EMG amplitude to the maximum computed over all conditions in individual participants^27^. Unless otherwise noted, the downstream analysis was performed regarding data during walking at *Fr* ~ 0.25.

### Extracting modules using matrix factorization

A data matrix was created by the processed EMGs, which consisted of 12 muscles × 200 time points in each condition. Each muscle vector in the data matrix was normalized to the unit variance, thus ensuring that the activity in all muscles was equally weighed. The unit variance was removed after module extraction to restore the original scaling^64^. Spatial modules and temporal modules in each condition were extracted from the EMG data matrix using an algorithm of nonnegative matrix factorization (NMF)^20,24,33^. NMF assumes that a muscle activation pattern ***M*** in a given time period comprises a linear combination of a few spatial modules, ${{\varvec{w}}}_{n}^{SP}$, and temporal modules, ${{\varvec{c}}}_{n}^{TE}$. Therefore, the (*i*, *j*)th element of the EMG data matrix ***M***_*i,j*_ is represented as follows:

$${{\varvec{M}}}_{i,j}=\sum_{n=1}^{N}{{\varvec{w}}}_{i,n}^{SP}{{\varvec{c}}}_{n,j}^{TE}+\varepsilon ({{\varvec{w}}}_{in}\ge 0, {{\varvec{c}}}_{nj}\ge 0)$$

where we specify the relative contributions of the muscles involved in the module, *n*. *N* denotes the number of modules. *ε* is the residual. To prevent local minima, the extraction procedure was repeated 20 times, each time with *W* and *C* initiated with different uniformly distributed random values between 0 and 1. The solution yielding the highest VAF value was selected for the analysis^65^.

To select the number of spatial and temporal modules needed for data reconstruction, between 1 and 12 modules were extracted from the EMG data matrices. The goodness-of-fit of the data reconstruction was quantified for each number of modules by the average of the variability accounted for (VAF), which was defined as 100 × the coefficient of determination from the uncentred Pearson’s correlation coefficient^66,67^. At each number of modules, we performed cross-validation^43^. In the cross-validation procedure, the EMG data matrices were first divided into five equal partitions. Modules were extracted from the pooled data set that consisted of four of the five partitions. These modules were fit to the remaining unused partition by only updating *C* with fixed *W* using the NMF algorithm, and the goodness of fit was quantified as the VAF value. This cross-validation procedure was repeated 20 times at each number of modules, each time with different randomly selected data partitions. The number of modules was determined to be the minimum number required for a VAF of > 90%.

### Clustering modules

To facilitate comparison of the spatial modules among the different gravity levels, the spatial modules identified from all participants in each gravity level were classified into a set of clusters using an unsupervised classification, k-means clustering, according to the cosine of the angle between the pair of vectors, i.e., cosine similarity, of the coordination patterns in the 12-dimensional Euclidean space^65^. If more than two spatial modules extracted from a given participant were classified into the same cluster, the modules that denoted the longer Euclidean distance from the centroid of the cluster were excluded from the analysis. The cluster centroids were defined as the representative spatial modules (SPc modules) in each gravity level. These processes were performed to enhance the focus on features of the modules regarding gravity by minimizing the intersubject variability of the extracted modules. Consecutively, clustering was further implemented on the SPc modules of all gravity levels. Here, we used k-means clustering with pairwise constraints; the SPc modules in the same gravity level cannot be classified into the same cluster^68^. We calculated the silhouette values of each classification while changing the number of clusters from 2 to 10 to define the optimal number of clusters as one that yielded the largest mean silhouette value across all SPc modules^69^. The series of clustering procedures was analogously followed for the comparison of the temporal modules among the different gravity levels. The cluster centroids were defined as the representative temporal modules (TEc modules) in each gravity level. Before each of the classifications, the spatial and temporal modules were normalized such that the individual muscle-weighting vector and temporal coefficient vector were the unit vector.

### Extracting modules using tensor decomposition

Matrix factorization of the EMG data enabled us to identify the differences in spatial and temporal modules in each gravity level, whereas how the spatial and temporal modules were modulated depending on the gravity levels could not be clearly demonstrated. Therefore, a tensor decomposition model (CANDECOMP/PARAFAC; CP) was fit to the EMG data to quantify the task-dependent variable in addition to the spatial and temporal (ST) modules^34,35^. For the tensor decomposition, the EMG data at *Fr* ~ 0.25 were arranged in a 3-dimensional array comprising 12 muscles × 200 time points × 5 gravity levels across all participants (i.e., 45). The CP model assumes that a muscle activation pattern *P* comprises a linear combination of a few spatial modules, ${{\varvec{w}}}_{n}^{ST}$, and temporal modules, ${{\varvec{c}}}_{n}^{ST}$, with a task-dependent variable, *t*_*n*_. Therefore, the (*i*, *j, k*)th elements of the 3-dimentional EMG data array *P* are represented as follows:

$${{\varvec{P}}}_{i,j,k}=\sum_{n=1}^{N}{\lambda }_{n}{{\varvec{w}}}_{i,n}^{ST}{{\varvec{c}}}_{j,n}^{ST}{{\varvec{t}}}_{k,n}+\varepsilon ({{\varvec{w}}}_{i,n}\ge 0, {{\varvec{c}}}_{j,n}\ge 0, {{\varvec{t}}}_{k,n}\ge 0)$$

where the relative contributions of the module, *n*, are specified. *N* denotes the number of modules. *ε* is the residual.${\lambda }_{n}\ge 0$ represents the scaling factor for the combination of the *n*th spatial and temporal modules and the task-dependent variables of the *n*th ST modules under the conditions ${{{\varvec{w}}}_{n}^{T}{\varvec{w}}}_{n}=1$, ${{{\varvec{c}}}_{n}^{T}{\varvec{c}}}_{n}=1$, and ${{{\varvec{t}}}_{n}^{T}{\varvec{t}}}_{n}=1$. The parameters were estimated to minimize the sum-of-squared error between the original and the reconstructed EMG data under nonnegative constraints for ***w***, ***c*** and ***t***. We relied on the tensor toolbox in MATLAB^70,71^. The function “cp_nmu” was used for the analysis.

Furthermore, the modulation of spatial and temporal modules was investigated in relation to the treadmill speed at which the 3-dimensional EMG data array comprised 12 muscles × 200 time points × [5 gravity levels × 7 treadmill speeds across all participants (i.e., 315)]. The CP model was also applied to the EMG data to quantify the task-dependent variables depending on both the gravity conditions and walking speeds with prescribed spatial and temporal modules. The number of modules shown in the main results was determined as that corresponding to the maximum number of SPc and TEc modules.

### Testing the similarities and differences in modules

The similarities of muscle-weighting vectors in the SPc modules were calculated between the module with the gravity of 1 g and the module classified into the same cluster in each of the remaining gravity levels based on the cosine similarity^25^. Thus, a cosine similarity closer to 1 indicated a greater similarity in the directions of the two vectors. The similarity of activation coefficients in the TEc modules was also quantified based on the correlation coefficient^26,37^. Moreover, the similarities of modules between a gravity of 1 g and each of the remaining gravity levels were calculated across all pairs of modules that were matched to each other by maximizing the cosine similarity between each spatial module pair and the correlation coefficient between each temporal module pair^65^. The overall similarity of modules was quantified as the median value of the cosine similarity and the correlation coefficient across all module pairs and participants.

To identify the similarity between the SPc/TEc modules and the spatial/temporal components of the ST modules, we modelled the SPc and TEc modules as a linear combination of the spatial components and temporal components of ST modules, respectively, as follows:

$${{\varvec{w}}}_{n}^{SP}=\sum_{h=1}^{N}{{\varvec{f}}}_{n,h}^{SP}{{\varvec{w}}}_{h}^{ST}+\varepsilon$$

$${{\varvec{c}}}_{n}^{TE}=\sum_{h=1}^{N}{{\varvec{f}}}_{n,h}^{TE}{{\varvec{c}}}_{h}^{ST}+\varepsilon$$

where ${{\varvec{w}}}_{n}^{SP}$ and ${{\varvec{c}}}_{n}^{TE}$ are the SP and TE modules, respectively, and ${{\varvec{w}}}_{h}^{ST}$ and ${{\varvec{c}}}_{h}^{ST}$ are the spatial and temporal components of the ST modules, respectively^27,72^. *ε* is the residual. The coefficients ${{\varvec{f}}}_{n,h}^{SP}$ and ${{\varvec{f}}}_{n,h}^{TE}$ ≥ 0 specify how the *n*th SPc and TEc modules, respectively, are fractionated into the *h*th component of ST modules. The coefficients ${{\varvec{f}}}_{n,h}^{SP}$ and ${{\varvec{f}}}_{n,h}^{TE}$ were identified using nonnegative least squares. The goodness-of-fit was quantified between the original SPc and TEc modules, ${{\varvec{w}}}_{n}^{SP}$ and ${{\varvec{c}}}_{n}^{TE}$, respectively, and the reconstructed modules as the VAF value.

### Statistics

Normality in the data were assessed using Shapiro–Wilk tests (*p* > 0.05). If the assumption of the normality was not confirmed, a non-parametric test was used.

The difference in VAF values among conditions and the numbers of modules was tested using two-way repeated-measures ANOVA. After selecting the number of modules, post hoc pairwise comparisons were performed with Bonferroni’s method to test the difference in VAF values among the gravity levels regarding the selected number of modules.

The differences in the muscle-weighting vectors and activation coefficient vectors among the different gravity levels and muscles were also tested using two-way repeated-measures ANOVA. Post hoc pairwise comparisons were performed with Bonferroni’s method to test the differences in the vectors among the gravity levels. To test the differences in activation coefficient vectors, a gait cycle normalized to 200 time points was equally divided into 10 intervals and averaged in each interval; 10 points in total were used for the statistical analysis. Moreover, a non-parametric Friedman’s test with a post hoc test was performed to test the difference in the overall cosine similarity and correlation coefficient between pairs of each gravity levels.

One-way repeated-measures ANOVA was performed to test the differences in task-dependent variables of each ST module among the gravity levels at *Fr* ~ 0.25. Post hoc pairwise comparisons were applied with Bonferroni’s method. Post hoc power analyses for the ANOVA were performed using G*Power^73^. The differences in task-dependent variables were also tested among the gravity levels and treadmill speeds using two-way repeated-measures ANOVA.

The confidence interval for all similarity tests was estimated using a bootstrapping procedure with 10,000 rounds of resampling of muscle-weighting vectors and activation coefficients and recalculating the cosine similarity, correlation coefficient, or VAF value across each pair of vectors^74^. A chance level was defined as the lower bounds of the 95% confidence interval.

## Supplementary Information

Supplementary Information.

## Data Availability

The data that support the findings of this study are available from the corresponding author upon reasonable request.
