# Supplementary material for: Modulation of spatial and temporal modules in lower limb muscle activations during walking with simulated reduced gravity
Source: Sci Rep. 2021 Jul 20;11:14749. doi: 10.1038/s41598-021-94201-9 (PMC8292403; doi:10.1038/s41598-021-94201-9)
Supplement: Supplementary file 1 — Supplementary Information. [file 41598_2021_94201_MOESM1_ESM.pdf]

Supporting information

Title: Modulation of spatial and temporal modules in lower limb muscle activations during walking with simulated reduced gravity

Authors: Shota Hagio[1,2], Makoto Nakazato[1], Motoki Kouzaki[1,2]

Author Affiliations:

[1]Laboratory of Neurophysiology, Graduate School of Human and Environmental Studies, Kyoto University, Kyoto 606-8501, Japan

[2]Unit of Synergetic Studies for Space, Kyoto University, Kyoto 606-8502, Japan

Supporting Table 1. Two-way repeated-measures ANOVA tables for the effects of muscles and gravity levels on the task-dependent variables of SP modules

SP_1		*df*	*F*	*p*	*partial* η^2
	muscle	11, 55	37.34	< 0.001	0.88
	gravity	4, 20	3.33	0.030	0.40
	muscle*gravity	44, 220	3.65	< 0.001	0.42

SP_2		*df*	*F*	*p*	*partial* η^2
	muscle	11, 33	31.77	< 0.001	0.91
	gravity	4, 12	1.18	0.37	0.28
	muscle*gravity	44, 132	2.76	< 0.001	0.48

SP_3		*df*	*F*	*p*	*partial* η^2
	muscle	11, 66	48.00	< 0.001	0.89
	gravity	4, 24	2.61	0.061	0.30
	muscle*gravity	44, 264	3.32	< 0.001	0.36

SP_4		*df*	*F*	*p*	*partial* η^2
	muscle	11, 33	14.73	< 0.001	0.83
	gravity	3, 9	0.23	0.074	0.28
	muscle*gravity	33, 99	1.63	0.034	0.35

Supporting Table 2. Two-way repeated-measures ANOVA tables for the effects of phase and gravity levels on the task-dependent variables of TE modules

TE_1		*df*	*F*	*p*	*partial* η^2
	phase	9, 54	84.62	< 0.001	0.93
	gravity	4, 24	2.28	0.090	0.28
	phase*gravity	36, 216	2.25	< 0.001	0.27

TE_2		*df*	*F*	*p*	*partial* η^2
	phase	9, 18	51.01	< 0.001	0.96
	gravity	3, 6	3.82	0.077	0.66
	phase*gravity	27, 54	0.50	0.97	0.20

TE_3		*df*	*F*	*p*	*partial* η^2
	phase	9, 63	6.82	< 0.001	0.49
	gravity	4, 28	3.26	0.026	0.32
	phase*gravity	36, 252	3.25	< 0.001	0.32

TE_4		*df*	*F*	*p*	*partial* η^2
	phase	9, 63	115.59	< 0.001	0.94
	gravity	3, 21	5.11	0.0082	0.42
	phase*gravity	27, 189	4.46	< 0.001	0.39

Supporting Table 3. Two-way repeated-measures ANOVA tables for the effects of gravity levels and walking speeds on the task-dependent variables of ST modules

ST_1		*df*	*F*	*p*	*partial* η^2
	speed	6, 48	103.87	< 0.001	0.93
	gravity	4, 32	13.71	< 0.001	0.63
	speed*gravity	24, 192	3.06	< 0.001	0.28

ST_2		*df*	*F*	*p*	*partial* η^2
	speed	6, 48	22.45	< 0.001	0.74
	gravity	4, 32	28.65	< 0.001	0.78
	speed*gravity	24, 192	6.47	< 0.001	0.45

ST_3		*df*	*F*	*p*	*partial* η^2
	speed	6, 48	14.05	< 0.001	0.64
	gravity	4, 32	48.50	< 0.001	0.86
	speed*gravity	24, 192	13.50	< 0.001	0.63

ST_4		*df*	*F*	*p*	*partial* η^2
	speed	6, 48	95.62	< 0.001	0.92
	gravity	4, 32	19.95	< 0.001	0.71
	speed*gravity	24, 192	20.80	< 0.001	0.72

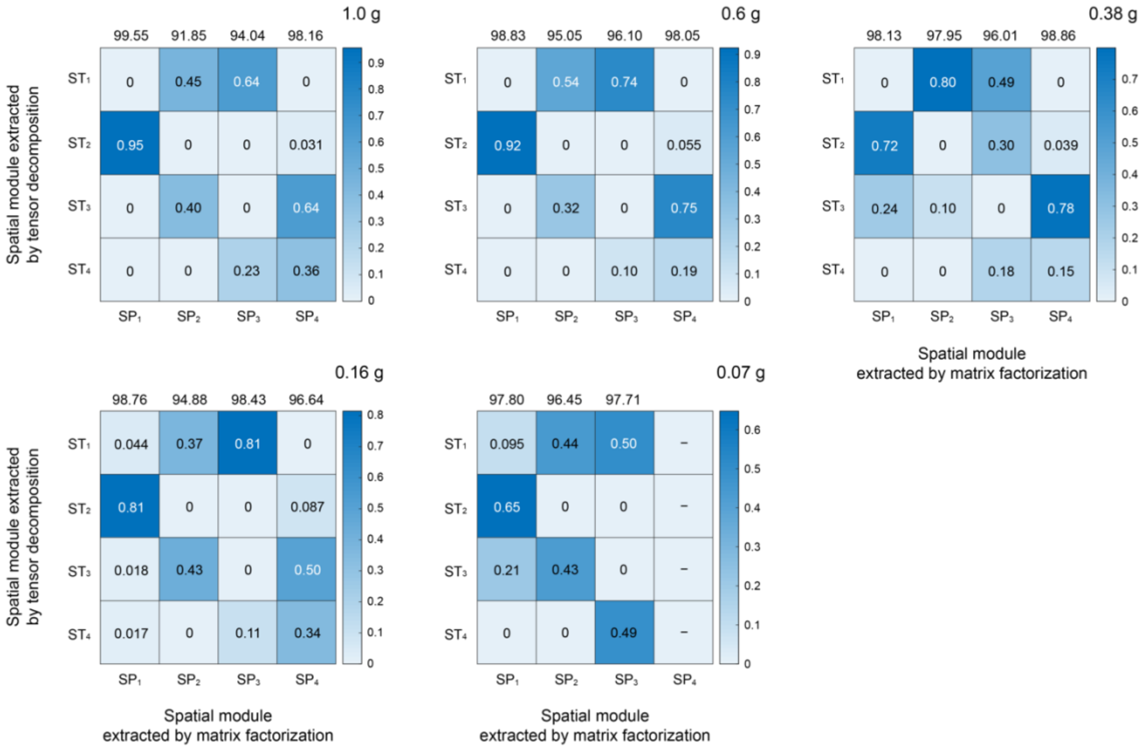

Supporting figure 1. Coefficients of the linear regression model for spatial modules. The coefficients of the linear model that explains the spatial (SP) modules as a linear combination of the spatial components of spatiotemporal (ST) modules are shown. The SP and ST modules correspond to those in Figs. 4 and 7, respectively. The values shown above each panel indicate the data variability accounted for by the linear model.

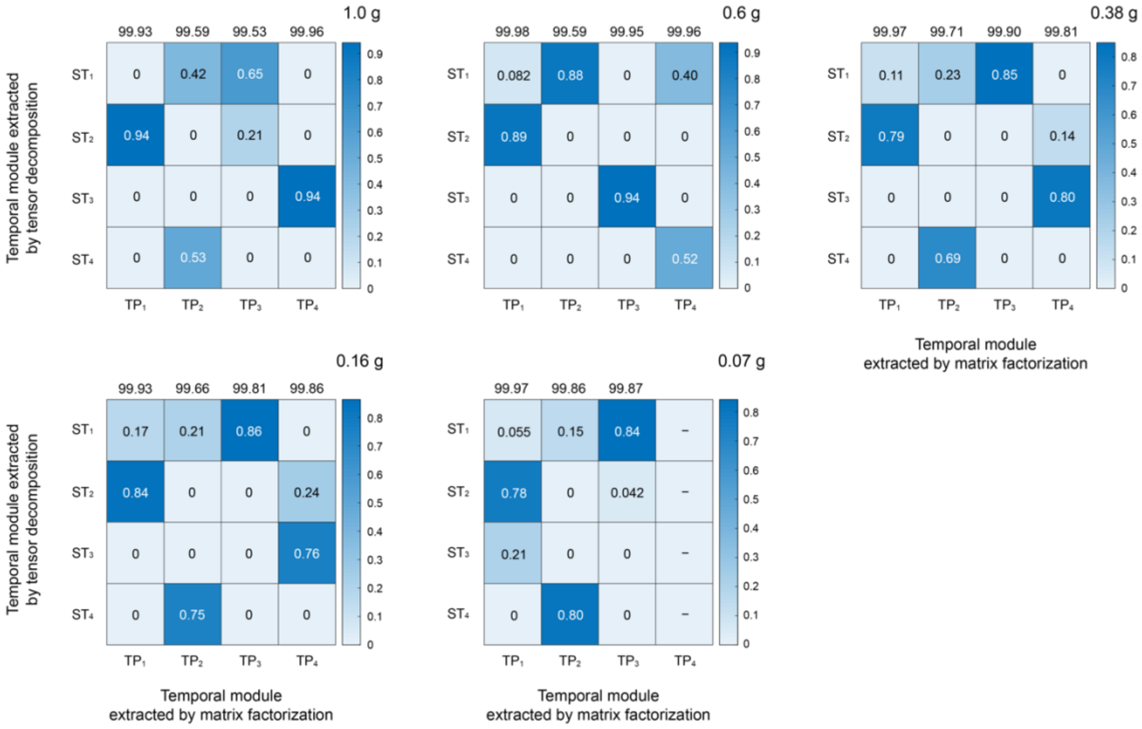

Supporting figure 2. Coefficients of the linear regression model for temporal modules. The coefficients of the linear model that explains the temporal (TP) modules as a linear combination of the temporal components of ST modules are shown. The TP and ST modules correspond to those in Figs. 5 and 7, respectively. The values shown above each panel indicate the data variability accounted for by the linear model.

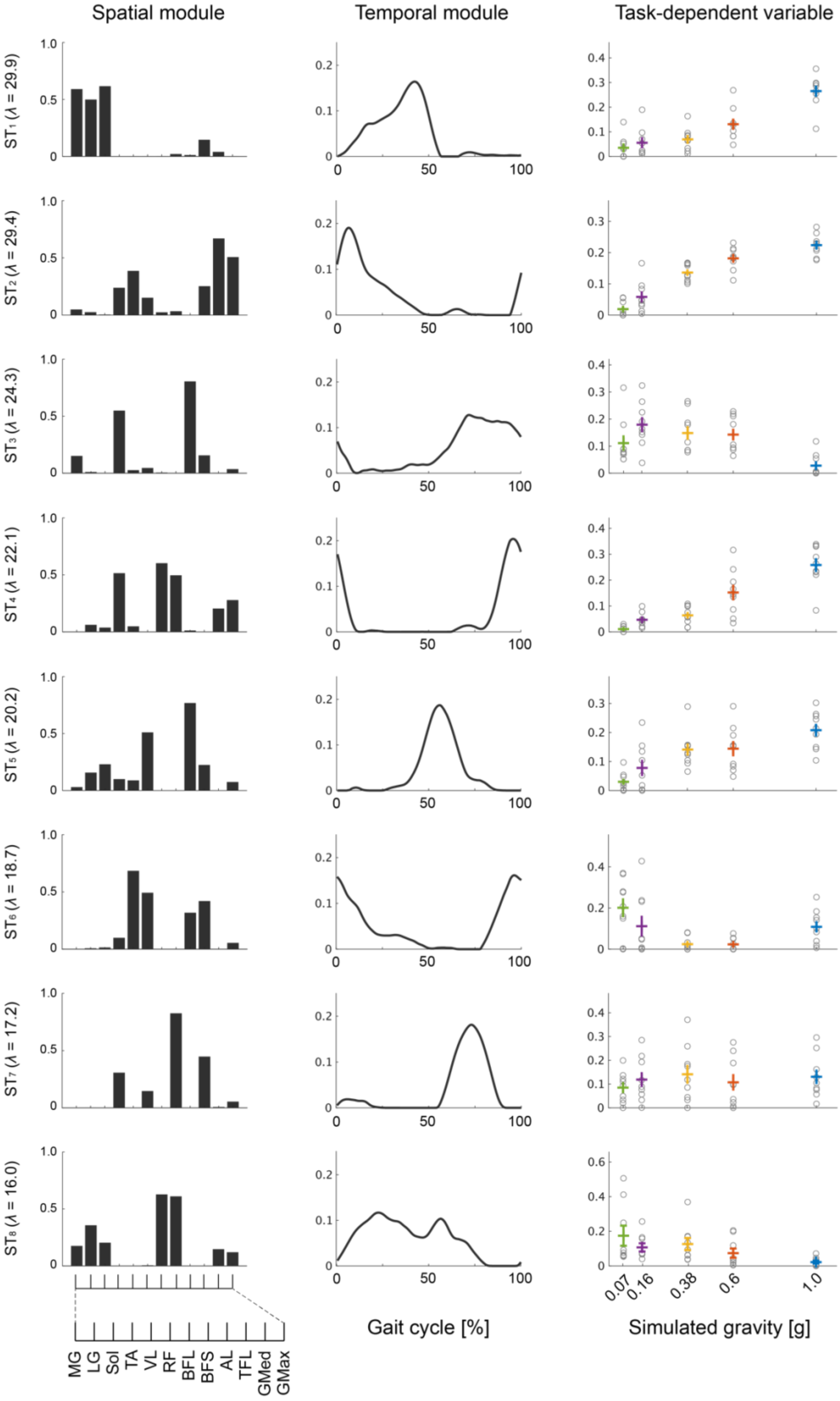
Spatial module
Temporal module
Task-dependent variable
ST1 (λ = 29.9)
ST2 (λ = 29.4)
ST3 (λ = 24.3)
ST4 (λ = 22.1)
ST5 (λ = 20.2)
ST6 (λ = 18.7)
ST7 (λ = 17.2)
ST8 (λ = 16.0)
MG LG Sol TA VL RF BFL BFS AL TFL GMed GMax
Gait cycle [%]
Simulated gravity [g]
0.07 0.16 0.38 0.6 1.0

Supporting figure 3. Spatiotemporal module and task-dependent variable.

Spatial (left) and temporal (middle) modules and corresponding task-dependent variables (right) extracted from the EMG data tensor at *Fr* ~ 0.25 are shown. The number of modules was determined to be the minimum number required for a VAF of > 60%. λ denotes the scaling factor. Each circle of the task-dependent variables indicates the data for individual participants. The mean and standard error of the means are represented as the horizontal and vertical lines of a coloured crossed line in each gravity level.

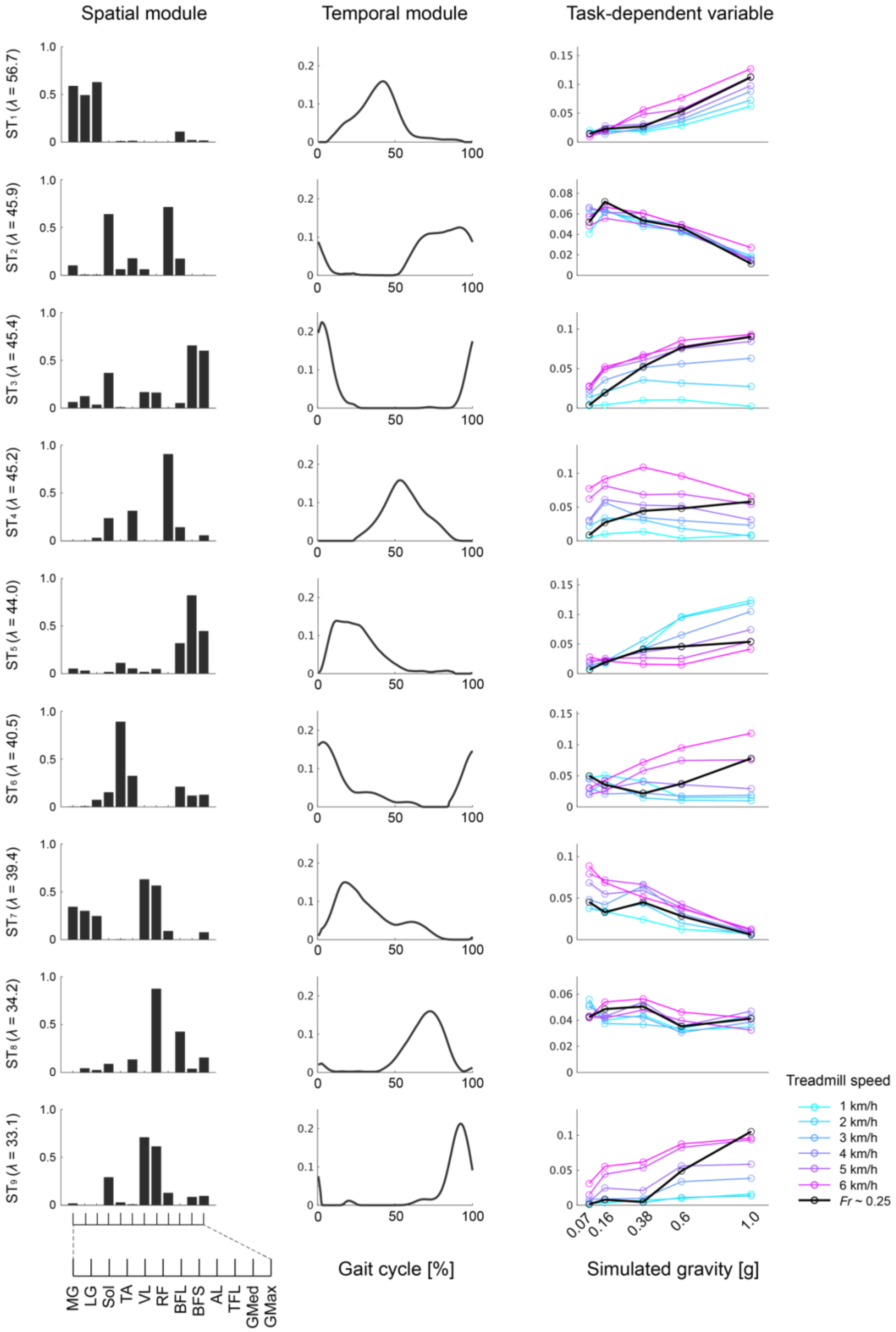

Spatial module
Temporal module
Task-dependent variable
ST1 (λ = 56.7)
ST2 (λ = 45.9)
ST3 (λ = 45.4)
ST4 (λ = 45.2)
ST5 (λ = 44.0)
ST6 (λ = 40.5)
ST7 (λ = 39.4)
ST8 (λ = 34.2)
ST9 (λ = 33.1)
MG
LG
Sol
TA
VL
RF
BFL
BFS
AL
TFL
GMed
GMax
Gait cycle [%]
Simulated gravity [g]
Treadmill speed
1 km/h
2 km/h
3 km/h
4 km/h
5 km/h
6 km/h
Fr ~ 0.25

Supporting figure 4. Spatiotemporal module and task-dependent variable across walking speeds.

Spatial (left) and temporal (middle) modules and corresponding task-dependent variables (right) extracted from the EMG data tensor are shown. The number of modules was determined to be the minimum number required for a VAF of > 60%. λ denotes the scaling factor. The circles and lines for the task-dependent variables in the individual treadmill speeds (1-6 km/h and speed determined by the Froude number (*Fr*) corresponding to 0.25) are distinguished by colour.

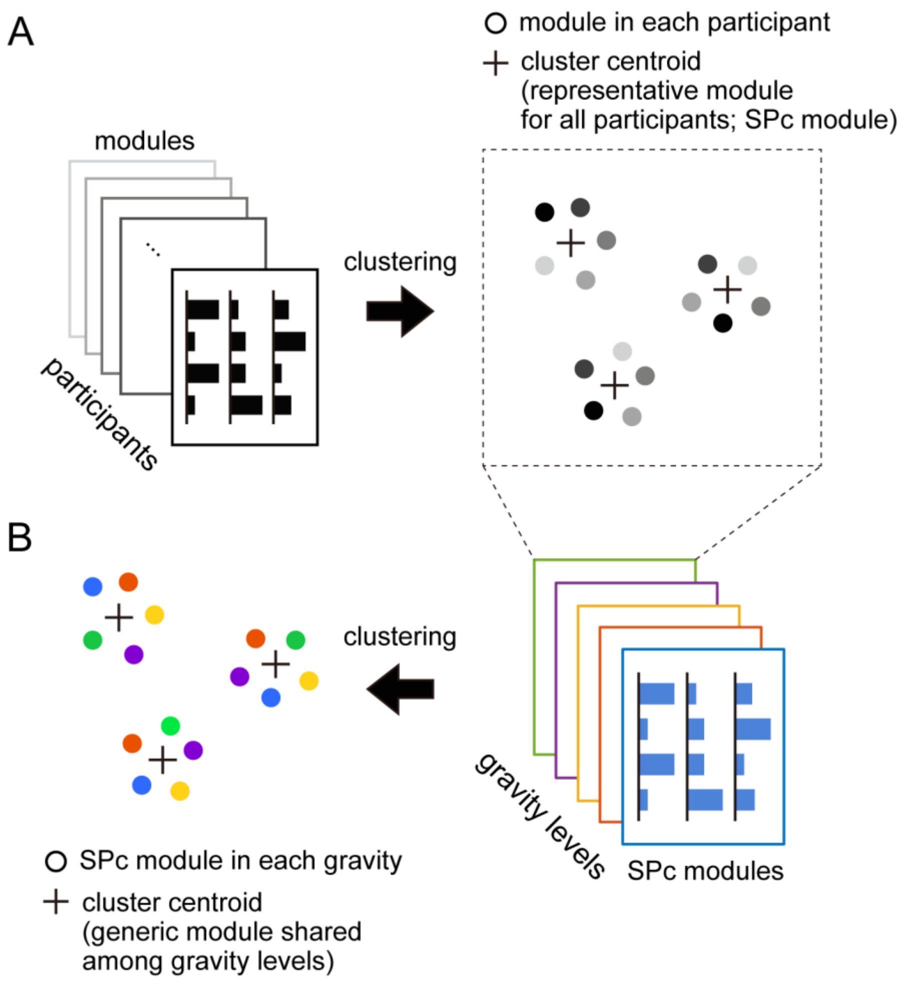

Supporting figure 5. Schematic diagrams of clustering spatial modules.

A: Spatial modules, i.e., muscle weighting vectors (shown as bar graphs), identified from all participants in each gravity level were classified into a set of clusters using k-means clustering. For display purposes, the muscle weighting vectors distributed in high-dimensional space were simplified as 2-dimensional representation (coloured circles corresponding to the modules in each participant shown in a left panel) in the right panel. The cluster centroids (+) were defined as the representative spatial modules (SPc modules) in each gravity level. *B*: Clustering was further implemented on the SPc

modules of all gravity levels. The vectors of the SPc modules in high-dimensional space were represented in 2 dimensions (coloured circles corresponding to the modules in each gravity are shown in the right panel) illustrated in the left panel. The cluster centroids (+) were defined as the generic spatial modules shared among all gravity levels. The series of clustering procedures was analogously followed for the comparison of the temporal modules among the different gravity levels.
